# Beyond the report: a qualitative exploration of safety incidents in maternity services

**DOI:** 10.1136/bmjoq-2025-004020

**Published:** 2026-03-31

**Authors:** Emma Beecham, Gráinne Brady, Paulina Bondaronek, James O’Carroll, Dimitrios Siassakos, Stephanie Glaser, Katie Gilchrist, Jenny Dorey, Rebecca Knagg, Cecilia Vindrola-Padros

**Affiliations:** 1 Department of Targeted Intervention, University College London, London, UK; 2 Institute of Health Informatics, University College London, London, UK; 3 University College London Hospitals NHS Foundation Trust, London, UK; 4 Institute for Women’s Health, University College London, London, UK

**Keywords:** Obstetrics and gynecology, Qualitative research, Patient safety

## Abstract

**Background:**

Maternal and neonatal mortality in the UK remains high, underscoring safety concerns in maternity care. Incident reporting remains a key mechanism for identifying risks and driving improvement, yet challenges, including underreporting and limited organisational learning, persist.

**Aim:**

The primary aim of the study was to explore clinicians’ preferences and behaviours in maternity patient safety reporting within a tertiary hospital.

**Methods:**

We conducted a two-phase qualitative study in a UK tertiary teaching hospital maternity service. Phase 1 involved AI-supported Big Qualitative (Big Qual) thematic analysis (using Caplena and Infranodus) of the first 400 patient safety incident reports submitted via the local electronic reporting system over a 5-month period (June–November 2024). Phase 2 comprised semistructured interviews with 14 maternity clinicians conducted between April and June 2025 and informed by phase 1 findings. Interview data were analysed using a Rapid Assessment Procedure and framework-based thematic analysis. Findings from both phases were integrated at the interpretation stage to examine reporting practices, barriers and enablers and opportunities for organisational learning, drawing on sociotechnical systems and safety-II-informed concepts.

**Results:**

Thematic analysis of incident reports identified ten recurrent topics including staffing capacity, documentation discrepancies and communication issues. Interviews highlighted barriers such as psychological safety, form complexity and limited feedback, alongside enablers including visible learning and supportive leadership. Inconsistencies in reporting behaviours, feedback mechanisms and system integration were evident, with underreporting of near misses and staff conduct identified as key gaps.

**Conclusions:**

This study offers a nuanced view on how incident reporting is enacted in practice within maternity care. By combining interview data with Big Qual incident analysis, it identifies actionable insights for improving safety and organisational learning. Recommendations include simplifying reporting systems, embedding psychological safety, standardising processes and enhancing feedback and cross-professional learning.

**WHAT IS ALREADY KNOWN ON THIS TOPIC**

- Incident reporting is central to improving maternity safety, yet underreporting, inconsistent feedback and limited organisational learning persist across UK services. Existing research rarely explores how digital reporting systems and cultural factors such as hierarchy and psychological safety influence reporting behaviour in maternity care.

**WHAT THIS STUDY ADDS**

- This study combines AI-supported analysis of 400 incident reports with clinician interviews to reveal practical barriers, including system complexity, time pressure and limited feedback, as well as enablers such as supportive leadership and visible learning, while also highlighting underreported areas such as near misses and staff conduct.

**HOW THIS STUDY MIGHT AFFECT RESEARCH, PRACTICE OR POLICY**

- Findings can inform the design of more user-friendly, learning-oriented reporting systems by simplifying processes, embedding psychological safety and strengthening feedback and team learning. These insights align with the Patient Safety Incident Response Framework principles and demonstrate how combining AI-supported Big Qual analysis with qualitative methods can generate actionable, system-level learning for safer care.

## Introduction

### Background and rationale

 Maternity care in the UK is facing a critical juncture. Recent national data reveal a troubling escalation in maternal mortality, rising from 9.71 to 13.41 deaths per 100 000 maternities between 2022 and 2023.^1^ However, rates of stillbirth and neonatal death have shown modest declines, according to The Mothers and Babies: Reducing Risk through Audits and Confidential Enquiries across the UK and Office for National Statistics data, though persistent inequalities remain.^2 3^ These trends underscore the urgent need for systemic reform and targeted action to address safety gaps in maternity care.

In maternity services, incident reporting is an important tool for identifying safety risks to inform improvements in care delivery.^4^ However, reporting practices vary widely across settings, reflecting differences in organisational culture, system design and staff engagement.^5^ Despite the increasing adoption of electronic reporting systems, ongoing challenges remain, including underreporting of adverse events, limited feedback mechanisms and inadequate organisational learning.^6^

A recent systematic review identified a diverse global landscape of incident reporting practices, with studies describing both traditional and electronic systems.^6^ Key barriers to effective reporting included unsupportive organisational cultures, time constraints and limitations in system design. Notable gaps persist, particularly in the underreporting of near misses, absence of structured feedback mechanisms and limited consideration of staff experiences. These findings highlight the need for implementing consistent and accessible reporting systems that facilitate accurate and timely documentation of safety incidents.

However, to date, a minority of studies utilised electronic reporting systems with none reflecting the systems currently implemented in UK hospitals, or Patient Safety Incident Response Framework (PSIRF).^7^ While PSIRF offers a national framework for learning from harm, its implementation across maternity units remains variable.^8^ In addition, variability in incident reporting and review practices across UK maternity units has been reported.^9 10^

This study sought to examine incident reporting practices in maternity care, focusing on cultural and contextual influences. It explores how reporting is enacted within a maternity service, identifies practical barriers and enablers, and highlights opportunities for system-level improvement. It also summarises patient safety activities and presents key themes from a Big Qualitative (Big Qual) analysis of incident reports.

### Aim and research questions

### Aim

The primary aim of the study was to explore clinicians’ behaviours and preferences in maternity patient safety reporting within a tertiary hospital. Secondary aims included identifying gaps in current reporting practices and highlighting areas for improvement to inform recommendations for strengthening incident reporting systems.

### Research questions

1. How do clinicians in maternity services describe their behaviours and preferences with incident reporting, and what factors influence these practices?
2. What gaps and inconsistencies exist in current maternity incident reporting?
3. What opportunities for improvement can be identified to strengthen reporting systems and organisational learning?

## Methods

For the purposes of this study, an incident was defined as any reported event or circumstance within maternity care that resulted in, or had the potential to result in, unintended or unnecessary harm to a woman, baby or staff member, in line with national patient safety guidance.^7 11^ A near miss was defined as an event or circumstance that did not result in harm but had the potential to do so if not intercepted or mitigated. The term escalation was used to describe the process by which a concern, risk or event was communicated beyond the immediate clinical context, including formal reporting, referral to senior clinicians or activation of organisational safety and governance processes.

### Study design

We conducted a two-phase sequential qualitative study combining Big Qual analysis of incident reports (phase 1) with semistructured interviews (phase 2) to explore how maternity staff experience and engage with safety reporting, capturing both system-level patterns and individual perspectives. We selected a sample of the first 400 incident reports submitted in a tertiary hospital maternity service, via the local incident reporting system (InPhase, Buckinghamshire, UK) over a 5-month period (June to November 2024). These reports were selected consecutively to capture real-world reporting as it naturally occurred, thereby minimising selection bias that could arise from retrospective inclusion. The 5-month window was chosen to reflect variation in routine operational pressures, ensuring the data represent typical workflow conditions rather than exceptional periods. This study used two AI-supported tools for Big Qual analysis: Caplena (v2, AG, Zurich, Switzerland) for thematic analysis using Natural Language Processing and machine learning, and Infranodus (v5, Nodus Labs, Ways, Leeds, UK) for discourse mapping via network analysis and knowledge graphs. To enrich and contextualise these findings, we conducted semistructured interviews with 14 clinicians routinely working within maternity services to offer insight into staff perceptions, behaviours and barriers to reporting. Insights from the phase 1 Big Qual analysis informed the development of the interview guide, ensuring that interviews explored reporting thresholds, system usability, feedback mechanisms and incident types that appeared prominent or under-represented in the incident report data. The interviews were also designed to probe areas that were notably absent or inconsistently documented in the incident reports, such as near misses, staff behaviour, equity-related concerns and relational dynamics between staff, to better understand the reasons for these gaps and how reporting practices are shaped in everyday clinical work. The data synthesis was informed by sociotechnical systems and safety-II concepts,^6^ to capture the interplay between technology, clinical environments and human decision-making, and to support a learning-oriented interpretation of safety.

By analysing incident reports as sociotechnical artefacts rather than objective accounts of care, this study demonstrates how reporting systems both reflect and reinforce particular models of safety. AI-supported Big Qual analysis enables large-scale pattern recognition within these artefacts, but it cannot overcome the epistemic limits of the data itself. Understanding what incident reports systematically exclude is therefore as important as analysing what they contain.

The study was conducted and reported according to Standards for Reporting Qualitative Research guidance^12^ (online supplemental appendix 1).

#### Researcher reflexivity

As non-clinical researchers, we approached the study with curiosity and a systems-focused lens, informed by prior work including a systematic review in this area. Our outsider status often prompted clinicians to explain clinical jargon and embedded practices more explicitly, which enriched the interviews and also shaped our interpretation. We recognise that our positionality, lacking direct clinical experience, may have influenced which aspects of the reports we noticed or emphasised. To address this, we engaged in ongoing reflexive discussions within the research team, critically examining how our backgrounds, assumptions and prior knowledge might affect data interpretation and ensuring that multiple perspectives informed the analysis.

### Big Qual analysis

Patient safety and quality improvement have benefited from Big Qual methods, which involve large-scale qualitative datasets (typically 100+ participants^13^) that support thematic saturation, subgroup comparisons and integration with quantitative findings.^14 15^

Anonymous incident report data from the incident reporting system were exported as Comma-Separated Values files and analysed in both AI platforms. Infranodus was used first to surface key concepts, structural gaps and discourse patterns, which then informed the configuration of Caplena and the development of final thematic codes. Using both tools allowed for complementary insights, Caplena provided scalable thematic clustering, while Infranodus revealed deeper relational structures and emergent narratives within the data.

Caplena generated an initial theme set from 400 incident reports, which was independently reviewed by three researchers (EB, GB and CV-P) replicating the thematic analysis methods used in Martin *et al*.^16^ Caplena uses a transfer learning approach in which pretrained language models are adapted to new analytic contexts rather than applied as fixed classifiers. Although the underlying model has been trained on healthcare text from other clinical domains, its application in this study involved domain-specific refinement through iterative human-in-the-loop coding of maternity patient safety incident reports. Researchers reviewed and adjusted emerging themes across repeated cycles, ensuring that topic structures reflected the language and context of maternity incident reporting rather than patterns from the original training data. Consequently, the AI-supported analysis functioned as an assistive tool to surface patterns, with interpretive judgement retained by the research team.

We refined the coding framework by merging, removing or relabelling themes for clarity (EB). In parallel, Infranodus identified discourse patterns and thematic relationships, informing further refinements to the Caplena coding framework. To ensure consistency, two additional researchers reviewed 20% of the coded data (GB and CV-P).

Caplena and Infranodus follow strict EU-compliant privacy policies, retaining data only for the project’s duration and deleting them 6 months after completion. Analyses were conducted within University College London’s secure server, with data use limited to this research.

### Interviews

#### Sampling strategy

The interviews were carried out with a purposive sample of clinicians across labour ward, obstetric theatres, antenatal and postnatal locations. The sampling was guided by a sampling framework designed to recruit participants from different areas of maternity services and levels of seniority. Recruitment materials were circulated across professional groups and explicitly encouraged participation from clinicians with diverse professional, cultural and linguistic backgrounds. While the sample was self-selecting, purposive efforts were made to include clinicians from ethnic minority backgrounds and those for whom English was not a first language, recognising the relevance of equity and implicit bias in maternity safety and incident reporting. Detailed demographic data were not collected or reported due to the small sample size and potential risk of participant identifiability within the local context.

#### Context

The study was conducted in a tertiary teaching hospital located within an urban catchment area. As a regional referral centre, the hospital manages complex maternity cases, making it a rich setting for examining the nuances of incident reporting across varied clinical scenarios and patient populations.

#### Recruitment

Clinicians were identified through dissemination of project information via posters, group messaging platforms, and email lists, as well as through in‑person visits by the research team to maternity wards to introduce the project to staff. Interested staff shared their email addresses and were invited via email to participate in a semistructured interview between April and May 2025. The inclusion criterion was employment as a clinician within maternity services at the hospital. Participants were incentivised with a £20 gift voucher to participate. A self-selecting sample was recruited via an advertisement inviting individuals who wished to share their experiences of incident reporting. Nineteen clinicians expressed interest via email. Fourteen clinicians were consented to and participated in an interview.

#### Interview guide development and semistructured interviews

The interview guide was informed by the study’s aims, a recent systematic review,^6^ Big Qual analysis of 400 incident reports, and input from our academic, clinical, and Patient and Public Involvement and Engagement (PPIE) team. The PPIE team reviewed the draft for clarity and relevance, and their feedback shaped the final version (see online supplemental appendix 3). Semistructured interviews with clinicians were conducted via Microsoft Teams (V.25275.2601.4002.2815) or in person between April and June 2025, audio recorded with consent, and supplemented by interviewer notes. Recordings were transcribed for analysis.

#### Thematic analysis

Interview notes were summarised using RREAL (Rapid Evaluation and Appraisal Lab) Rapid Assessment Procedure sheets^17^ to identify early themes and support iterative analysis. The team familiarised themselves with the data and developed a coding framework, which was applied to transcripts and charted in Microsoft Excel (version 1808) for cross-case comparison.^18^ Guided by research questions and open to inductive insights, the framework is detailed in online supplemental appendix 4. Charting by two researchers (EB and GB) informed analysis of category interconnections and overarching patterns. This approach supported timely, team-based analysis while preserving contextual nuance.

#### Triangulation

Triangulation was achieved by comparing codes and emerging themes across the different datasets (Big Qual data and interview transcripts) and by integrating insights from Caplena and Infranodus. Final themes were validated through iterative team discussions, in which the coding framework and theme interpretations were critically reviewed, refined and agreed on. Additional validation was provided by incorporating feedback from PPIE contributors, ensuring that the final themes were robust, credible and reflective of multiple perspectives.

#### Patient and public involvement and engagement

A PPIE group of three members with lived maternity experience was involved throughout, contributing to the previous study, interview guide, interpretation of findings and dissemination. Three clinicians (midwifery, obstetrics, anaesthesia) also shaped recruitment, co-developed the guide and supported analysis. Their involvement ensured relevance, rigour and co-produced insights to enhance the study’s impact.

## Results

This section presents findings from two complementary data sources: (1) AI-supported Big Qual analysis of 400 maternity incident reports and (2) semistructured interviews with maternity clinicians.

### Thematic findings from incident reports: Caplena and Infranodus

**Figure 1:**
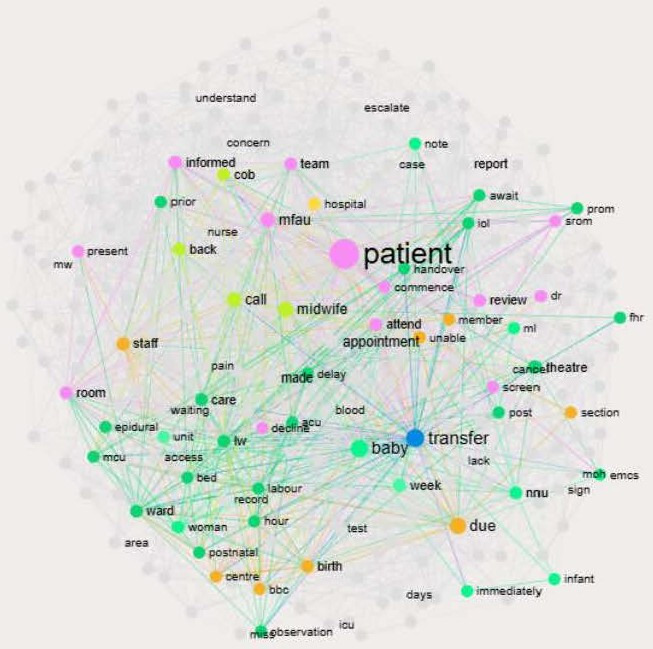
Network representation of the ‘transfer (logistics)’ topic identified through InfraNodus analysis of maternity incident reports.

One subtheme was removed during code cross-checking, indicating strong inter-coder agreement. See box 1 for an overview of the coding framework (a comparison of Caplena’s original coding and the final framework is provided in the online supplemental appendix 2). Infranodus also used GPT (Generative Pre-trained Transformer)-based models to generate descriptive summaries and visualise thematic structure through network graphs (see figure 1).

The analysis of 400 incidents using Caplena and Infranodus led to the identification of 10 recurrent topics (see box 1).

Box 1 **Thematic categories and illustrative subthemes from incident report analysis**

1. Patient care (*including subthemes of staffing capacity issues, transfer logistics and communication issues).
2. Documentation/system issues (*including Induction of labour proforma, risk assessment, information technology failure and electronic health record).
3. Delivery (*including VBAC, emergency caesarean and instrument delivery).
4. Professionals (*including staff conduct, multidisciplinary team working and social services/safeguarding team involved).
5. Interventions (*including epidural, induction and glucose tolerance testing).
6. Maternal factors (*including gestational diabetes, medical history and pre-eclampsia).
7. Environment (*including out of hours, homebirth and facilities/bed issues).
8. Monitoring (*including CTG monitoring, fetal doppler monitoring and observations).
9. Outcome (*including maternal death, neonatal death and readmission).
10. Neonatal factors (*including bradycardia, breech and shoulder dystocia).

*CTG, cardiotocography; VBAC, vaginal birth after caesarean.

**Figure 2:**
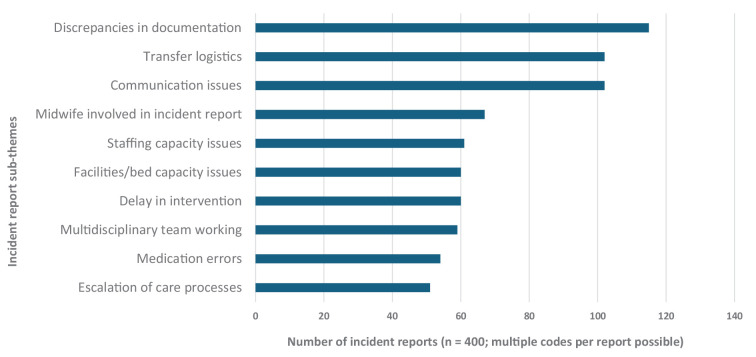
The top 10 most coded topics/subthemes from the analysis of 400 InPhase incidents in Caplena.

Figure 2 is a bar chart of the most frequently coded maternity patient safety incident report subthemes identified through AI-supported Big Qual thematic analysis. The chart demonstrates that documentation discrepancies (115 reports; 29%), communication issues (102; 26%) and transfer logistics (102; 26%) were the most commonly reported concerns. Medication errors, escalation of care processes and multidisciplinary team (MDT) working appeared less frequently. When interpreted alongside clinician interview data, these patterns highlight how system design, reporting thresholds and perceived psychological safety shape what types of incidents are most visible within reporting systems, providing early insight into areas of both concentrated risk and potential underreporting that informed subsequent qualitative exploration. Incidents may be coded under multiple topics, which explains why totals exceed 400.

Caplena also provides the following summary of the analysis of all 400 incidents: Respondents report frequent delays and disruptions in patient care due to staff shortages, capacity issues and administrative errors, affecting timely admissions, transfers, discharges and availability of essential services like ultrasounds and specialist appointments. There are multiple clinical safety incidents such as medication errors, missed follow-ups, poor handovers, procedural complications (eg, major obstetric haemorrhage, shoulder dystocia, neonatal resuscitation) and documentation lapses, which contribute to patient and staff distress, complaints and sometimes adverse outcomes. Additionally, infrastructure failures and interpersonal conflicts, ranging from IT breakdowns and equipment inadequacies to abuse towards staff and communication breakdowns, further compromise patient experience and safety.

Figure 2 presents the network graph and semantic structure of the ‘Transfer (logistics)’ topic derived from AI-supported InfraNodus analysis of 400 maternity incident reports. Transfer logistics was selected as an illustrative case because of its high prevalence and sociotechnical complexity, which lends itself to semantic network analysis.

Nodes represent words extracted from incident narratives, with node size indicating relational centrality based on co-occurrence with other terms rather than raw frequency, and links representing words appearing in close textual proximity. The prominence of terms such as patient, baby, transfer, delay and handover highlights how transfer-related incidents are framed around coordination across teams, timing and continuity of care. By revealing clusters of closely connected concepts, the figure illustrates how logistical challenges intersect with communication and staffing pressures, informing the study’s findings that transfer processes function as critical sociotechnical points of risk and are central to understanding how incidents are reported and interpreted within maternity services. Infranodus’ OpenAI’s GPT model generated the following summary using the main topics and keywords from the graph: The Transfer logistics—The timing of transfers between wards, theatres, labour and post-care units is critical for maintaining seamless patient care. Delays in transfer can lead to bottlenecks and stress on both staff and patients, underscoring the need for well-coordinated transitions that are mindful of care demands and labour dynamics.

### Interview findings

14 clinicians participated in a semistructured interview. These included: seven midwives from bands 5–7, four anaesthetists, two obstetricians and one nurse. 10 were female and four were male, covering antenatal, postnatal and labour wards. Thematic analysis identified six overarching domains:

#### Reporting practices and processes

There was evidence of a cultural shift towards learning, supported by frameworks such as PSIRF, which encouraged near-miss reporting. However, hierarchical dynamics and interpersonal sensitivities often led to informal handling of incidents involving colleagues. Reporting was primarily conducted via the local reporting system, InPhase, supplemented by protocols, trigger lists and duty of candour processes. Time pressures and workload influenced the timing and likelihood of reporting, and practices varied widely, with some staff relying on informal verbal reporting. As one anaesthetist explained:

(it’s) not very standardised as to what happens on the ground.

#### Feedback and organisational learning

Feedback mechanisms included email alerts, governance meetings and safety briefings, though consistency varied by department and role. Some clinicians voiced frustration over generic responses (eg, ‘we are recruiting’) that failed to address core concerns. Emotional support was offered through After-Action Reviews and clinical review groups, but access depended on leadership presence. Staff appreciated structured learning tools including safety newsletters and ‘Big 4’ briefings (daily huddles focused on four key operational priorities to enhance awareness and coordination across maternity services) yet called for more consistent follow-up. As one midwife noted:

I have filled in quite a lot (of incident reports) over the time and I often have made recommendations in the space that says what recommendations would you suggest…I don’t ever recall getting any response back from that

#### Barriers and enablers to reporting

Key barriers included time constraints, complex reporting forms, login issues and lack of protected time. Psychological safety remained a concern, with non-anonymity in the reporting system discouraging openness, particularly around staff behaviour. Uncertainty about reporting thresholds and role responsibilities led to duplication or omission. Conversely, visible impact and constructive feedback were strong motivators, as were supportive leadership, accessible systems and regular training. Clinicians appreciated flexibility in how and to whom they could report, and valued prompts embedded in protocols. Another midwife reflected on the impact of time constraints and performance-driven pressures, stating:

Just I think if managers could recognise that this takes time. …It’s not looking at whether you spent that extra time with somebody who is an asylum seeker. To use language line to give really good care.

#### Patient reporting and system transitions

Participants described challenges associated with transitions between incident reporting platforms, particularly in relation to continuity of data, loss of historical context and uncertainty about how incidents were tracked across systems. These transitions were perceived to disrupt organisational learning by fragmenting datasets and weakening feedback loops, rather than by altering who was able to submit reports. While patient reporting was not available within either platform, this was not identified by participants as a primary driver of reporting behaviour.

One participant described a perception of consistent transparency in incident handling.

We don’t hide them (incidents), we do a best interest meeting for the patients, which happen quite often…they (the patients) feel they’re being valued

However, this account is presented as an individual viewpoint rather than a generalisable claim. When considered alongside the wider literature on patient safety and organisational openness, this perception highlights variability in how transparency is experienced and understood by staff, rather than evidencing uniformly transparent practice.

#### Current strengths, gaps and opportunities for system improvement

Maternity services demonstrated a strong reporting culture, primarily led by midwives. Staff valued visible learning but identified persistent gaps in training, delegation and data capture. The local incident reporting system was perceived as cluttered, contributing to reporting fatigue and inconsistent follow-up, particularly for low-harm events. A lack of structured mechanisms to track outcomes or facilitate shared learning meant that reporting often relied on informal handovers.

Interviewees proposed several improvements: enhancing accessibility through mobile integration, simplified forms, anonymous reporting options and clearer ownership of reports; strengthening feedback processes with emotional support, EPIC systems electronic healthcare record integration and prompts aligned with PSIRF principles; and embedding team-based learning into handovers, simulations and interprofessional collaboration to foster trust and engagement. One anaesthetist saw the annual MDT learning day as a chance to strengthen team relations:

It’s a simulation-based training where anaesthetists, midwives and obstetricians participate all together… a unique opportunity to interact with each other and build some foundations.

#### Under-reported incident types and populations

Several incident types were routinely underreported, including postpartum haemorrhage, neonatal admissions and near misses, particularly when outcomes were favourable. Staff-related concerns such as bullying and interpersonal conflict were often omitted due to fear of escalation, especially among junior staff. Communication-related risks affecting non-English speakers were inconsistently documented, reflecting uncertainty around reporting procedures despite recognition of their clinical relevance. As one anaesthetist explained:

I think sometimes the non-English speaking patients possibly could be underreported purely because sometimes the communication lapse can be really difficult and we don’t have many language lines

## Discussion

### Interpretation of findings in the context of the wider literature

This study explored clinicians’ preferences and behaviours in maternity incident reporting, supported by Big Qual analysis of 400 incident reports and qualitative interviews with 14 clinicians. The findings reveal a complex interplay between cultural, procedural and systemic factors that shape reporting behaviours, feedback mechanisms and opportunities for improvement.

This study used triangulation not to validate one dataset against another, but to examine how different forms of safety data illuminate distinct aspects of incident reporting practice. Interviews captured clinicians’ accounts of how reporting is understood, enacted and negotiated in everyday work, while incident reports were analysed as artefacts of reporting shaped by system design, reporting thresholds, organisational expectations and time pressures. Rather than treating incident reports as direct representations of care quality, we examined how their content, structure and omissions reflect reporting behaviour. Integration occurred at the interpretive level, with interviews contextualising patterns in incident reports and incident data highlighting discrepancies between formal reporting outputs and clinicians’ described practices.

## Triangulation of data sources and synthesis of key insights

### Theme 1: reporting thresholds and what becomes reportable

Interviews revealed considerable variation in how clinicians interpreted reporting thresholds, particularly for near misses, staff conduct and events without immediate harm. These uncertainties were reflected in the incident report dataset, where reports overwhelmingly focused on tangible clinical processes (eg, transfers, documentation, monitoring) and adverse outcomes, with comparatively little narrative attention to interpersonal dynamics or near-miss recovery.

This pattern suggests that reporting systems and organisational norms implicitly privilege events that align with safety-I logics of error and failure, while adaptive actions, successful recoveries and relational risks remain less visible. Incident reports therefore function less as comprehensive safety narratives and more as filtered representations shaped by what staff perceive as legitimate, defensible or worth the time required to document.

These findings align with existing literature showing that incident reporting is shaped by subjective judgements about severity, legitimacy and blame, particularly for near misses and relational concerns.^19 20^ Prior studies have documented similar variability in reporting thresholds, but often rely primarily on interview data.^21^ By combining clinician accounts with large-scale analysis of incident report narratives, this study extends existing work by demonstrating how these judgements are materially enacted within reporting datasets, and how Big Qual methods can surface systematic patterns of omission that might otherwise remain anecdotal.

### Theme 2: system design and the shaping of narrative content

Clinicians consistently described reporting systems as time-consuming, cognitively burdensome and difficult to navigate, particularly during periods of high workload. These experiences were mirrored in the incident report analysis, which showed a predominance of short, task-focused descriptions and repeated completion of mandatory fields related to documentation, transfers and monitoring. Rather than indicating that these domains are inherently the most safety-critical, this pattern likely reflects how system architecture structures attention and narrative effort. Mandatory fields, drop-down menus and predefined categories appear to shape what is recorded, while more complex issues, such as communication breakdowns linked to hierarchy or language barriers, are less consistently articulated.^22^ Incident reports thus reveal how reporting systems actively co-produce safety knowledge, as the system design directly influences how events are interpreted, narrated and rendered analysable.^23^

### Theme 3: psychological safety, attribution and omission

Interviews highlighted ongoing concerns about psychological safety, especially in incidents involving colleagues, senior staff or interpersonal conflict. Fear of blame, escalation or damaging relationships often led to informal resolution rather than formal reporting. Consistent with this, the incident report dataset contained limited explicit reference to staff behaviour, conflict or discriminatory dynamics, despite interviewees identifying these as clinically and emotionally significant. This absence does not imply rarity but reflects the social risks of documenting such events in non-anonymous systems. In this way, incident reports reveal not only what is reported but also what is systematically difficult to record. Fear of adverse consequences, including blame, is a commonly identified barrier to incident reporting in patient safety literature.^24^ Our findings show these concerns produce systematic omissions in reports, with Big Qual revealing gaps that reflect structurally constrained reporting rather than incidental absence.

### Theme 4: feedback and organisational learning

Interviewees described feedback on incident reports as inconsistent, often limited to generic responses, with frustration around a lack of follow-up or clarity about next steps. This created uncertainty about how reported issues were addressed or whether they contributed to broader organisational learning. These accounts reflect a largely procedural approach to reporting, leaving clinicians with limited visibility into any actions or improvements arising from their reports. Reporting effort often outpaces perceived organisational learning, with sparse documentation of outcomes mirroring clinicians’ perceptions of weak feedback loops (eg, lack of feedback and weak loops in incident reporting systems).^6^ In the context of PSIRF, this highlights the potential of combining interview and report analysis to rapidly evaluate learning infrastructures.

### Theme 5: equity and differential visibility of risk

Interviews highlighted concerns about underreporting of incidents affecting non-English-speaking patients and those with complex social needs, often linked to uncertainty about how to categorise communication barriers or cultural factors in reporting systems. Incident reports similarly showed limited engagement with equity-related aspects of care, suggesting existing structures may lack the prompts, language or psychological safety needed to surface these risks. This aligns with evidence that safety reporting systems can reproduce systemic blind spots unless equity is explicitly built into reporting frameworks.^25^ Emerging literature in maternity safety shows how implicit and explicit provider bias shapes communication, responsiveness and escalation, contributing to differential risk and harm, particularly for marginalised groups. Because these harms are often relational, cumulative and normalised within everyday practice, they are unlikely to be readily captured within conventional incident reporting systems.^26^ Our findings suggest current frameworks may inadvertently perpetuate inequities by failing to prompt or legitimise documentation of communication barriers and social complexity. Using a combined analytic approach, we identified these blind spots in both clinician narratives and reporting data, demonstrating how rapid, mixed qualitative methods can support equity-informed safety analysis even in the absence of detailed demographic data.

**Figure 3:**
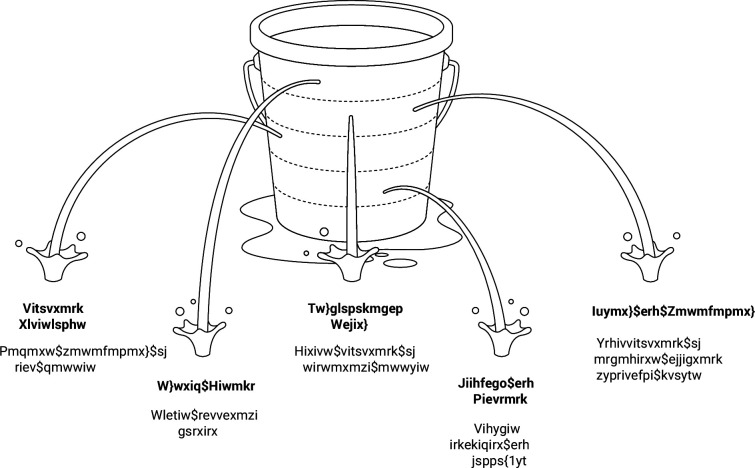
Integrated triangulation of incident report analysis and clinician interview findings on maternity incident reporting (diagram generated using NAPKIN AI (https://www.napkin.ai/).

Figure 3 demonstrates a visual representation of the triangulated themes. System-level features of incident reporting intersect with clinicians’ experiences of psychological safety, equity and the perceived value of reporting, shaping what incidents are reported and how they are framed. It highlights that underreporting in maternity care arises from interconnected sociotechnical and organisational factors, rather than individual behaviour, limiting learning from near misses, staff conduct issues and incidents affecting vulnerable groups.

This study is informed by sociotechnical systems theory and Safety-II concepts to support a learning-oriented interpretation of maternity incident reporting. However, the reporting systems under study remain predominantly organised around safety-I assumptions, privileging the documentation of errors, deviations and adverse outcomes. The analysis therefore examines incident reports as constrained sociotechnical artefacts, shaped by system design, organisational norms and prevailing safety logics. Safety-II concepts are used interpretively to examine what remains underrepresented, such as adaptive work, recovery and relational dynamics, rather than as an organising analytic framework. This plural and explicit positioning allows critical examination of both what reporting systems make visible and what they systematically obscure, without overstating the epistemic reach of incident data. Participants described a perceptible shift towards learning-oriented reporting, supported by frameworks such as PSIRF. However, this cultural shift remains uneven. Hierarchical dynamics and interpersonal sensitivities continue to influence reporting practices, particularly in cases involving colleagues. The reliance on informal verbal reporting and inconsistent use of the local reporting system suggests that while the infrastructure for learning exists, its operationalisation is fragmented. These findings echo existing literature highlighting the tension between formal systems and relational dynamics in clinical environments.^27 28^

Poorly managed incident reporting processes, particularly the absence of structured feedback and emotional support, have been shown to erode trust in organisational leadership, leading staff to seek validation and reassurance from colleagues instead.^29^ While this study focused on safety incidents, emerging approaches such as ‘Learning from Excellence’ offer complementary routes for reinforcing positive behaviours and psychological safety.^30^ The usability issues reported in the interviews reflect broader concerns about cognitive burden and interface design in clinical information systems, which have been shown to affect data quality and staff engagement.^31^

The incident analysis revealed a recurrent theme, identified by AI tools, of the transfer process being implicated in multiple safety events. This aligns with previous studies highlighting transfers and handovers as high-risk moments for adverse outcomes, particularly due to communication breakdowns and role ambiguity.^32 33^ These findings reinforce the need for structured handover protocols and clearer escalation pathways within maternity services.

The identification of underreported equity-related concerns, especially those affecting non-English-speaking patients, introduces an important and underexplored dimension to the existing literature. These findings also lend further weight to calls by Knight *et al*^34^ for the development of more inclusive patient safety frameworks that explicitly address communication barriers and the broader influence of social determinants of health. Recent evidence highlights systemic blind spots in maternity incident reporting, with ethnic disparities reflected in both the frequency and framing of reported events. Disproportionate representation of Black women in incident data, alongside differing root causes and patterns of communication failure, underscores the need for more inclusive, equity-sensitive approaches to safety analysis and governance.^35 36^ These findings also align with calls for greater patient involvement in safety governance, including co-produced incident reviews and culturally sensitive feedback mechanisms.^37^

By analysing incident reports as sociotechnical artefacts rather than proxies for care quality, this study demonstrates how AI-supported Big Qual analysis, combined with clinician interviews, can generate actionable insights into reporting practices and organisational learning in maternity services.

### Strengths and limitations

A key strength of this study is its analytical triangulation of interview data with large-scale incident report analysis, enabling examination of both lived reporting practices and their formal documentation. However, incident reports cannot be treated as neutral representations of safety events; they are shaped by system design, organisational culture and individual risk calculus.

The study is limited by its single-centre design and reliance on self-selected interview participants, whose accounts may reflect social desirability or recall bias. The AI-supported analysis inherits the constraints of the underlying reporting data and should be understood as amplifying, rather than correcting, existing patterns of visibility and omission within incident reporting systems. Finally, limiting the analysis to the first 400 consecutive incident reports provides a practical dataset but may have introduced bias and represents a study limitation.

### Implications for practice and policy

This study indicates that improving maternity incident reporting requires reframing reporting as a learning and sense-making practice rather than as an administrative mechanism for documenting harm alone. Incident reports are shaped by system design, psychological safety and locally negotiated reporting thresholds, and improvement efforts must therefore be co-designed with those who use reporting systems in practice to address these sociotechnical conditions and support meaningful quality improvement.

First, reporting systems should be redesigned through co-design with front-line clinicians to support meaningful narrative input while minimising avoidable cognitive burden. Simplification should focus on removing redundant fields, improving navigation and clearly distinguishing mandatory from optional content, while retaining sufficient space for free-text narrative. Mobile-accessible reporting enhancements, such as brief point-of-care reporting functions or deferred completion options that allow reports to be finalised after clinical episodes, may support timely documentation when developed collaboratively and aligned with existing clinical workflows.

Second, psychological safety should be treated as a core component of reporting infrastructure and addressed through participatory approaches. Co-designed interventions that support shared understanding of what constitutes reportable harm, near misses and staff conduct concerns may reduce uncertainty around reporting thresholds. These may include team-based reflection sessions, facilitated safety huddles, or structured prompts within reporting systems that normalise the reporting of near misses and relational harms alongside adverse outcomes.

Third, feedback mechanisms should be strengthened through explicit PSIRF-aligned learning loops that demonstrate how incident reports contribute to system change. Examples include tiered feedback processes where reporters receive acknowledgement and local learning summaries, teams receive aggregated thematic insights through regular governance or safety meetings, and organisations feedback resulting improvement actions. Integration of reporting data with PSIRF patient safety incident response plans, learning responses and quality improvement registers may enhance traceability between reporting, analysis and action.

Fourth, equity considerations must be explicitly embedded within reporting systems through co-design with staff and communities who support patients with additional needs. Structured prompts relating to communication barriers, language needs, or reasonable adjustments, alongside training on equitable reporting practices, may improve visibility of currently under-represented risks. Analytic approaches that routinely examine reporting patterns by patient characteristics can further support equity-informed quality improvement.

Finally, AI-supported Big Qual methods can support quality improvement when used as reflective tools within co-designed improvement cycles rather than as surveillance mechanisms. Their value lies in rapidly surfacing patterns, clusters and absences within reporting data to inform PSIRF-aligned prioritisation, particularly when combined with qualitative inquiry that examines both reported incidents and gaps in reporting practice.

### Future research

Future research should compare incident reporting across hospital settings to identify systemic versus local barriers. Equity-sensitive frameworks are needed to address language, disability and cultural factors. Longitudinal studies on incident reporting system transition could assess impacts on reporting, data quality and engagement. Exploring patient and family involvement in safety mechanisms like duty of candour and PALS may support more transparent, participatory maternity safety governance.

## Conclusions

This study identifies ongoing challenges and opportunities in maternity incident reporting, based on triangulated data from interviews, reports and system analysis. Reporting behaviours were shaped by time pressures, interpersonal dynamics and system usability, with clinicians often using informal methods. Inconsistent and emotionally unsupported feedback contributed to frustration and reduced trust. Barriers included psychological safety concerns, complex forms and unclear delegation; enablers included visible impact, supportive leadership and embedded prompts.

Improving reporting requires simpler, mobile-accessible systems integrated into clinical workflows. Anonymous options, clearer fields and embedded prompts can ease reporting fatigue. Cultural change is also key, fostering psychological safety, non-punitive responses and open dialogue. Stronger feedback loops, inclusive engagement and equity-sensitive design are essential for meaningful learning and safer care.

## Supplementary material

10.1136/bmjoq-2025-004020 online supplemental appendix 1

10.1136/bmjoq-2025-004020 online supplemental appendix 2

10.1136/bmjoq-2025-004020 online supplemental appendix 3

10.1136/bmjoq-2025-004020 online supplemental appendix 4

## Data Availability

All data relevant to the study are included in the article or uploaded as supplementary information.

## References

[1] Hasegawa K, Leis M, Howitt P (2024). Darzi a the national state of patient safety 2024: prioritising improvement efforts in a system under stress. https://www.imperial.ac.uk/Stories/National-State-Patient-Safety-2024.

[2] Page GL, Smith LK, Fenton AC (2025). MBRRACE-UK perinatal mortality surveillance: UK perinatal deaths of babies born in 2023 - state of the nation report. https://timms.le.ac.uk/mbrrace-uk-perinatal-mortality/surveillance/files/MBRRACE-UK-perinatal-mortality%20surveillance-report-2023.pdf.

[3] Office for National Statistics (2025). Child and infant mortality in England and Wales: 2023. https://www.ons.gov.uk/peoplepopulationandcommunity/birthsdeathsandmarriages/deaths/bulletins/childhoodinfantandperinatalmortalityinenglandandwales/2023.

[4] Hassan NAH, Rahman HA, Knights J (2024). Cultivating patient safety culture in midwifery practices through incident reporting. Br J Midwifery.

[5] Care Quality Commission (2024). National review of maternity services in England 2022 to 2024. https://www.cqc.org.uk/publications/maternity-services-2022-2024.

[6] Beecham E, Brady G, Iqbal S (2025). Systematic review of patient safety incident reporting practices in maternity care. BMJ Open Qual.

[7] NHS England (2022). Patient safety incident response framework (PSIRF). https://www.england.nhs.uk/patient-safety/patient-safety-insight/incident-response-framework.

[8] Healthcare Safety Investigation Branch H (2025). Investigating under the patient safety incident response framework (PSIRF): sharing HSSIB learning for future development. https://www.hssib.org.uk/patient-safety-investigations/investigating-under-the-patient-safety-incident-response-framework-psirf-sharing-hssib-learning-for-future-development/investigation-report.

[9] Shah A, Mohamed-Ahmed O, McClymont C (2014). Conditions triggering local incident reviews in UK hospital maternity units: A national survey. JRSM Open.

[10] Shah A, Mohamed-Ahmed O, Peirsegaele P (2015). Incident reviews in UK maternity units: a systematic appraisal of the quality of local guidelines. BMC Pregnancy Childbirth.

[11] World Health Organization (2009). Conceptual framework for the international classification for patient safety. https://iris.who.int/server/api/core/bitstreams/fc68f57e-3cf6-4fbf-a95d-2686593ea392/content.

[12] O’Brien BC, Harris IB, Beckman TJ (2014). Standards for reporting qualitative research: a synthesis of recommendations. Acad Med.

[13] Brower RL, Jones TB, Osborne-Lampkin LT (2019). Big Qual: Defining and Debating Qualitative Inquiry for Large Data Sets. Int J Qual Methods.

[14] Bates DW, Levine D, Syrowatka A (2021). The potential of artificial intelligence to improve patient safety: a scoping review. NPJ Digit Med.

[15] Davidson E, Edwards R, Jamieson L (2019). Big data, qualitative style: a breadth-and-depth method for working with large amounts of secondary qualitative data. *Qual Quant*.

[16] Martin S, Beecham E, Kursumovic E (2026). Comparing human vs. machine-assisted analysis to develop a new approach for Big Qualitative Data Analysis. *PLOS Digit Health*.

[17] Vindrola-Padros C, Chisnall G, Cooper S (2020). Carrying Out Rapid Qualitative Research During a Pandemic: Emerging Lessons From COVID-19. Qual Health Res.

[18] Gale NK, Heath G, Cameron E (2013). Using the framework method for the analysis of qualitative data in multi-disciplinary health research. BMC Med Res Methodol.

[19] Vincent C (2007). Incident Reporting and Patient Safety.

[20] Macrae C (2016). The problem with incident reporting. *BMJ Qual Saf*.

[21] Waring JJ (2004). A qualitative study of the intra-hospital variations in incident reporting. Int J Qual Health Care.

[22] Gong Y, Kang H, Wu X (2017). Enhancing Patient Safety Event Reporting. Appl Clin Inform.

[23] Dekker S (2017). The field guide to understanding’human error.

[24] Archer S, Hull L, Soukup T (2017). Development of a theoretical framework of factors affecting patient safety incident reporting: a theoretical review of the literature. BMJ Open.

[25] Goldman J, Rotteau L, Lo L (2026). Integrating equity into incident reporting and patient concerns systems: a critical interpretive synthesis. *BMJ Qual Saf*.

[26] Afulani PA, Ogolla BA, Oboke EN (2021). Understanding disparities in person-centred maternity care: the potential role of provider implicit and explicit bias. Health Policy Plan.

[27] Ginsburg L, Easterbrook A, Geerts A (2025). ‘We listened and supported and depended on each other’: a qualitative study of how leadership influences implementation of QI interventions. *BMJ Qual Saf*.

[28] Vincent C, Carthey J, Macrae C (2017). Safety analysis over time: seven major changes to adverse event investigation. Implementation Sci.

[29] Olagundoye V, Quinlan M, Burrow R (2022). Stress, anxiety, and erosion of trust: maternity staff experiences with incident management. *AJOG Glob Rep*.

[30] Kelly N, Blake S, Plunkett A (2016). Learning from excellence in healthcare: a new approach to incident reporting. Arch Dis Child.

[31] Cahill M, Cleary BJ, Cullinan S (2025). The influence of electronic health record design on usability and medication safety: systematic review. BMC Health Serv Res.

[32] Siassakos D, Fox R, Bristowe K (2013). What makes maternity teams effective and safe? Lessons from a series of research on teamwork, leadership and team training. Acta Obstet Gynecol Scand.

[33] McCarthy S, Motala A, Lawson E (2025). Use of structured handoff protocols for within-hospital unit transitions: a systematic review from Making Healthcare Safer IV. *BMJ Qual Saf*.

[34] Knight M, Bunch K, Kenyon S (2020). A national population‐based cohort study to investigate inequalities in maternal mortality in the United Kingdom, 2009‐17. Paediatr Perinat Epidemiol.

[35] Clark R, Klaiman T, Sliwinski K (2025). Communication failures and racial disparities in inpatient maternity care: a qualitative content analysis of incident reports. BMJ Open Quality.

[36] Farrant K, Faluyi D, Watson K (2022). Role of ethnicity in high-level obstetric clinical incidents: a review of cases from a large UK NHS maternity unit. BMJ Open Qual.

[37] Ocloo J, Matthews R (2016). From tokenism to empowerment: progressing patient and public involvement in healthcare improvement. *BMJ Qual Saf*.

