# Supplementary material for: Beyond the report: a qualitative exploration of safety incidents in maternity services
Source: BMJ Open Qual. 2026 Mar 31;15(1):e004020. doi: 10.1136/bmjoq-2025-004020 (PMC13052773; doi:10.1136/bmjoq-2025-004020)
Supplement: online supplemental appendix 2 [file bmjoq-15-1-s002.pdf]

Appendix 2

Comparison of original AI coding from Caplena and the final coding framework

**Original AI coding themes**	**Resulting themes from human validation and cross checking**
1. Other (including subthemes of information sharing, patient concerns and appointment scheduling)	1. Documentation/system issues (including Induction of labour proforma, risk assessment, IT failure and electronic health record)
2. Birth (including newborn transfer and retained placenta)	2. Delivery (including VBAC, Emergency caesarean and instrument delivery)
3. Care delays (including delayed care)	3. Outcome (including maternal death, Neonatal death and readmission)
4. Clinical (including induction of labour, caesarean section and epidural)	4. Interventions (including epidural, induction, and glucose tolerance testing)
5. Capacity (including NNU capacity and bed issue)	5. Environment (including out of hours, homebirth and facilities/bed issues)
6. Obstetrics (including 3rd degree tear, shoulder dystocia and uterine rupture)	6. Maternal factors (including gestational diabetes, medical history and pre-eclampsia)
7. Staffing (including staffing shortage)	7. Professionals (Staff conduct, Multidisciplinary team working and Social services/safeguarding team involved)
8. Patient safety (including patient discharged and infection control)	8. Monitoring (including CTG monitoring, fetal doppler monitoring and observations)
9. Complications (including pathological CTG and reduced fetal movements)	9. Neonatal factors (including bradycardia, breech, and shoulder dystocia)
10. Transfer (including referral to 30B6A and diversion)	10. Patient care (including subthemes of staffing capacity issues, transfer logistics, shared decision making and communication issues)
11. Medication (including vaccination and anti-D)	
12. Equipment (including bereavement suite and Entonox issues)	
