# Supplementary material for: Beyond the report: a qualitative exploration of safety incidents in maternity services
Source: BMJ Open Qual. 2026 Mar 31;15(1):e004020. doi: 10.1136/bmjoq-2025-004020 (PMC13052773; doi:10.1136/bmjoq-2025-004020)
Supplement: online supplemental appendix 3 [file bmjoq-15-1-s003.pdf]

Appendix 3-Interview schedule for clinicians in maternity services

Maternity Patient Safety-Interview Schedule for Clinicians in Maternity Services

- Introduce self and that we are conducting interviews to understand their experiences of patient safety incident reporting in maternity services
- Reassure we anticipate it should take about 30-45 minutes to run through all the questions
- Assure confidentiality that all of the information shared will be anonymised
- Thank participant for agreeing to contribute (no right or wrong answers; views and experiences are what matter)
- Ask if participant has any questions
- Check participant is happy for the interview to be audio recorded

Section 1: Current Incident Reporting Systems and Practices

1. **Can you start by telling me your role in maternity services?**

2. **Can you describe the current incident reporting systems used in maternity services at your hospital?**
    - Which systems do you use and how?
        - On phone/computer/paper?

3. **How do you usually report incidents?**
    - How would you define what an incident is?
    - What are the steps involved in the reporting process?
    - Do you feel confident identifying an incident?
    - Which incidents do you report? And why?
    - Which incidents do you not report? And why?
    - Do you report near misses?
    - Have you ever been reluctant to raise concerns, if so why?
    - Who is responsible for handling and reviewing the reports?

4. **How often do you report incidents?**
    - What is the typical timeframe for reporting an incident from the time it occurs?

5. **How do you receive feedback on the incidents you report?**
    - How often?

- Do you ever receive information about the changes that have been introduced following incident reporting?
- Do you receive feedback on incidents that have occurred elsewhere, that others have reported?
- How are you supported after the reporting of difficult or particularly emotional incidents?

6. **Facilitators and barriers:**
    - What facilitates the process of reporting incidents?
    - What are barriers to the process of reporting incidents?
    - How do these barriers affect the accuracy and comprehensiveness of incident reports?

7. **How do you advise patients about reporting incidents?**
    - If a patient expresses a concern about an incident that staff may have not been involved in, what do you advise them?
    - Do you feel confident to advise them on steps to take to report?
    - How are patients communicated with before, during and after an incident is raised?
    - What are the feedback mechanisms to report back to patients after they have reported an incident?

Section 2: Differences in Reporting Practices

8. **What do you think about the new system InPhase compared to the old system of Datix?**
    - What are the main differences in your eyes?
        - Functionality, usage and retrieval differences?
    - Do you see any improvements?
    - Do you see any decline in quality/accessibility?
    - How do they differ in terms of consequences/feedback after you have reported an incident?

9. **Are there any noticeable differences in the quality and consistency of data recorded in these systems?**
    - Is there any data consistently missing?

10. **How would you rate the ease of use of each system (Datix, InPhase)?**
    - Are there any features or aspects of these systems that you find particularly helpful or challenging?

11. **Have you been involved in escalated reporting for more severe incidents i.e.. incidents that get escalated to NHS England/Maternity and Newborn Safety Investigations (MNSI)?**
    - If yes, what is the level of detail required from you for these reports?
    - Is there anything you feel could be incorporated into InPhase that is required for the MNSI reports?

Section 3: Gaps in Reporting and Areas for Improvement

12. **Can you identify any gaps or limitations in the current incident reporting practices?**
    - What is currently working well?
    - What is currently not working well?
    - Are there specific types of incidents or details that are often underreported or overlooked?
    - Are there any patient groups where you feel incidents are less reported?

13. **What improvements do you think could be made to the current incident reporting systems or processes?**
    - How can your hospital better support clinicians in reporting incidents accurately and efficiently?
        - Training needs, system needs?
    - Better Feedback systems?
    - What changes could be made to incident reporting systems or processes to foster greater inclusivity and equity for both staff and patients?

14. **How do you think improving incident reporting practices could impact patient safety in maternity services?**
    - Are there any specific initiatives or technologies you believe could enhance reporting practices?

15. **Final Thoughts:** Do you have any additional comments or suggestions regarding incident reporting practices?

Thank the interviewee for their time and explain the next steps in the research process.
