# Supplementary material for: Beyond the report: a qualitative exploration of safety incidents in maternity services
Source: BMJ Open Qual. 2026 Mar 31;15(1):e004020. doi: 10.1136/bmjoq-2025-004020 (PMC13052773; doi:10.1136/bmjoq-2025-004020)
Supplement: online supplemental appendix 4 [file bmjoq-15-1-s004.pdf]

Appendix 4 - Interview analysis coding framework

Role	Current incident reporting systems used	Methods of reporting		Feedback mechanisms	Barriers to reporting	Facilitators to reporting	Patient reporting	Differences between reporting practices		Gaps or limitations in the current incident reporting practices			Suggested Improve-ments	How incident reporting impacts patient safety
		Definition	Steps of the process					Differences between Inphase and Datix	Differences in accuracy	Working well	Gaps	Under-reported		
